# Chitosan Nanoparticles Act as an Adjuvant to Promote both Th1 and Th2 Immune Responses Induced by Ovalbumin in Mice

**DOI:** 10.3390/md9061038

**Published:** 2011-06-14

**Authors:** Zheng-Shun Wen, Ying-Lei Xu, Xiao-Ting Zou, Zi-Rong Xu

**Affiliations:** Key Laboratory for Molecular Animal Nutrition of Ministry of Education, Feed Science Institute, College of Animal Science, Zhejiang University (Huajiachi Campus), Qiutao North Road 164, Hangzhou 310029, China; E-Mails: ashun789@yahoo.cn (Z.-S.W.); 106413708@qq.com (Y.-L.X.)

**Keywords:** chitosan nanoparticles, adjuvant, immune response, ovalbumin

## Abstract

The study was conducted to investigate the promoted immune response to ovalbumin in mice by chitosan nanoparticles (CNP) and its toxicity. CNP did not cause any mortality or side effects when mice were administered subcutaneously twice with a dose of 1.5 mg at 7-day intervals. Institute of Cancer Research (ICR) mice were immunized subcutaneously with 25 μg ovalbumin (OVA) alone or with 25 μg OVA dissolved in saline containing Quil A (10 μg), chitosan (CS) (50 μg) or CNP (12.5, 50 or 200 μg) on days 1 and 15. Two weeks after the secondary immunization, serum OVA-specific antibody titers, splenocyte proliferation, natural killer (NK) cell activity, and production and mRNA expression of cytokines from splenocytes were measured. The serum OVA-specific IgG, IgG1, IgG2a, and IgG2b antibody titers and Con A-, LPS-, and OVA-induced splenocyte proliferation were significantly enhanced by CNP (*P* < 0.05) as compared with OVA and CS groups. CNP also significantly promoted the production of Th1 (IL-2 and IFN-γ) and Th2 (IL-10) cytokines and up-regulated the mRNA expression of IL-2, IFN-γ and IL-10 cytokines in splenocytes from the immunized mice compared with OVA and CS groups. Besides, CNP remarkably increased the killing activities of NK cells activity (*P* < 0.05). The results suggested that CNP had a strong potential to increase both cellular and humoral immune responses and elicited a balanced Th1/Th2 response, and that CNP may be a safe and efficacious adjuvant candidate suitable for a wide spectrum of prophylactic and therapeutic vaccines.

## 1. Introduction

Vaccination remains the most effective and cost-efficient means to prevent infectious diseases. The latest trend towards novel and safer vaccines utilizes well-characterized antigens, like purified proteins, peptides, or carbohydrates. These so-called subunit vaccines enable the focusing of the immune response to the desired specificity without the risks associated with vaccines based on whole inactivated or live attenuated pathogens. Unfortunately, such subunit antigens are often poor immunogens when administered alone [1]. Therefore, an adjuvant is required to potentiate the immune response to the coadministrated antigen.

However, strong adjuvant activity is often correlated with increased toxicity and adverse effects. The unique capacity of the extract Quil A from the bark of *Quillaja saponaria* and its purified saponin QS-21 to stimulate both the Th1 immune response and the production of cytotoxic T-lymphocyte against exogenous antigens makes them ideal for use in subunit vaccines and vaccines directed against intracellular pathogens as well as for therapeutic cancer vaccines [2,3]. However, in addition to pain on injection, severe local reactions and granulomas, toxicity includes severe haemolysis [4–7] making such adjuvants unsuitable for human uses other than for life threatening diseases, such as HIV infection or cancer [8]. Freund’s complete adjuvant (FCA) remains amongst the most potent known adjuvants and a particularly powerful stimulant of both cellular and humoral immunities [9]. Unfortunately, FCA causes severe reactions and is too toxic for human use. Currently, aluminum compounds (Alum) is the only adjuvant in vaccines licensed by the Food and Drug Administration (FDA) for use in humans in the United States [10]. While Alum is safe, it is a relatively weak adjuvant, particularly when used with subunit antigens. Moreover, the Alum is a mild Th2 adjuvant that can effectively enhance IgG1 antibody responses, but it is rarely associated with Th1 type immune responses [11]. Furthermore, Alum is poor at stimulating cell-mediated immune responses, and may actively block activation and differentiation of cytotoxic T-lymphocytes [12]. Hence, there is a major unmet need for a safe and efficacious adjuvant capable of boosting cellular plus humoral immunity [13].

The ability of biodegradable microparticles to promote vaccine-specific immunity has been recognized for more than 80 years [14]. Early studies have demonstrated that the adjuvant potency may be amplified by the formation of nanoparticles with uptake by dendritic cells (DCs) [15,16], and this contributes to their enhancing effects on innate and antigen-specific cellular immunity [17]. Nanoparticles often exhibit significant adjuvant effects in parenteral vaccine delivery since they may be readily taken up by antigent presenting cells. The submicron size of nanoparticles allows them to be taken up by M-cells, in mucosa-associated lymphoid tissue (MALT), *i.e.*, gut-associated, nasal-associated and bronchus-associated lymphoid tissue, initiating sites of vigorous immunological responses [18]. However, the mechanism of action of particulate vaccine adjuvants is not fully understood [19], particularly for polymeric nanoparticles. Possible mechanisms have been suggested: that nanoparticles induce cytokine release by epithelial cells, shift the Th1/Th2 balance, activate macrophages and natural killer cells (NK) and improve the delayed-type hypersensitive reaction, increase cytotoxicity and induce mitosis in cells producing interleukins, breeding factors and interferon, or simply by increased absorption of antigen [20].

Chitosan is a natural nontoxic biopolymer produced by the deacetylation of chitin, a major component of the shells of crustaceans such as crab, shrimp, and crawfish. Recently, chitosan has received considerable attention for its commercial applications in the biomedical, food, and chemical industries [21–23]. The unique character of nanoparticles could make chitosan nanoparticles exhibit more superior activities than chitosan. Chitosan nanoparticles have been synthesized as drug and vaccine delivery carriers as reported in previous studies [24,25]. Due to their bioadhesive, biocompatibility, biodegradability and penetration-enhancement properties, chitosan nanoparticles are most efficiently taken up by phagocytotic cells inducing strong systemic and mucosal immune responses against antigens [20,26,27]. Besides enhancing the immune response by stimulating the uptake by phagocytotic cells, chitosan and its nanoparticles may also stimulate the immune system. Chitosan have been reported to have immune-stimulating activity such as increasing accumulation and activation of macrophage and polymorphonuclear cells, inducing cytokines after intravenous administration [28–33]. Therefore, the use of chitosan nano- and microparticles used as immunological adjuvants to induce both humoral and cell-mediated immunity is promising. However, the evaluation of chitosan nanoparticles as an adjuvant for subcutaneous vaccination has received less attention. Therefore, we hypothesized that chitosan nanoparticles (CNP) may have the adjuvant potential to amplify immune response against vaccination by stimulating the innate immune system. The present study was designed to evaluate the effect of CNP on the immune response induced by a model subunit antigen ovalbumin (OVA) in mice. OVA was used because this protein is considered to be an inert antigen with low capacity to modulate the immune response and is widely used as a model antigen. As a positive control, Quil A is known to be a potent adjuvant for experiment use.

## 2. Materials and Methods

### 2.1. Mice

Five-week-old female ICR mice (Grade II) weighing 18–22 g were purchased from Zhejiang Chinese Medical University Animal Research Center (Hangzhou, China) and acclimatized for one week prior to use. Rodent laboratory chow and tap water were provided *ad libitum* and maintained under controlled conditions with a temperature of 24 ± 1 °C, humidity of 50 ± 10%, and a 12/12 h light/dark cycle. All procedures related to the animals and their care conformed to the internationally accepted principles as found in the Guidelines for Keeping Experimental Animals issued by the government of China.

### 2.2. Chemicals and Cell Line

Chitosan (CS) was obtained from the Chitosan Company of Pan’an, Zhejiang, China (degree of deacetylation, 95%; average molecular weight, 220 kDa). Ovalbumin (OVA), concanavalin A (Con A), 3-(4,5-dimethylthiazol-2-yl)-2,5-diphenyltetrazolium bromide (MTT), lipopolysaccharide (LPS), RPMI-1640 medium, and rabbit anti-mouse IgG peroxidase conjugate were purchased from Sigma Chemical Co., Saint Louis, MO, USA; goat anti-mouse IgG1, IgG2a and IgG2b peroxidase conjugate were from Southern Biotech. Assoc., Birmingham, AL, USA; Quil A was kindly provided by BrenntagNordic A/S, Denmark. Fetal calf serum (FCS) was provided by Hangzhou Sijiqing Corp., Hangzhou, Zhejiang, China. Cytokines (IL-2, IL-10, IFN-γ) detecting ELISA kits were from Rapidbio Lab., West Hills, CA, USA. Trizol was from Invitrogen, China; revert Aid™ M-MuLV reverse transcriptase was from Fermentas, USA; diethylpyrocarbonate (DEPC) and ribonuclease inhibitor were from Biobasic, Canada; oligo (dT)_18_ were from Sangon, China.

Human leukemia K562 cell lines, sensitive to natural killer (NK) cells, were purchased from the Institute of Cell Biology, Chinese Academy Sciences, Shanghai, China. They were maintained in the logarithmic phase of growth in RPMI 1640 medium supplemented with 2 mM l-glutamine (Sigma), 100 IU/mL penicillin, 100 g/mL streptomycin (Sigma), and 10% fetal calf serum at 37 °C under humidified air with 5% CO_2_.

### 2.3. CNP Preparation and Characterization

Chitosan (CS) was obtained from the Chitosan Company of Pan’an (Zhejiang Province, China). The degree of deacetylation was about 95% as determined by elemental analysis, and the average molecular weight of the chitosan was 220 kDa as determined by viscometric methods [34]. Chitosan nanoparticles were prepared and characterized as previously described [35]. Briefly, Chitosan was dissolved at 0.5% (w/v) with 1% (v/v) acetic acid (HOAc) and then raised to pH 4.6–4.8 with 10 N NaOH. CNP were formed by coacervation between positively charged chitosan (0.5%, w/v) and negatively charged sodium tripolyphosphate (0.25%, w/v). Nanoparticles with different mean size were obtained by adjusting the volume ratio of chitosan to tripolyphosphate solution. Nanoparticles were purified by centrifugation at 9000 g for 30 min. Supernatants were discarded and chitosan nanoparticles were extensively rinsed with distilled water to remove any NaOH residues, and freeze dried before further use or analysis. The freeze-dried chitosan nanoparticles were suspended in water for characterization or use for other experiments. Particle size distribution and the zeta potential of chitosan nanoparticles were determined using Zetasizer Nano-ZS90 (Malvern Instruments). The analysis was performed at a scattering angle of 90°at a temperature of 25 °C using samples diluted to different concentration with de-ionized distilled water. Atomic force microscopy (AFM, SPM-9500J3) was used for visualization of the chitosan nanoparticles deposited on silicon substrates operating in the contact mode. AFM imaging was performed using Si_3_N_4_ probes with a spring constant of 0.06 N/m.

A stock CNP suspension or CS solution with a concentration of 3 mg/mL was prepared. The CNP was sterilized by passing it through a 0.22 μm Millipore filter, CS was autoclaved to remove any contaminant and then analyzed for endotoxin level by a gel-clot *Limulus* amebocyte lysate assay (Zhejiang A and C Biological, Zhejiang, China). The endotoxin level in the stock soln. was less than 0.5 EU/mL.

### 2.4. Toxicity Assays

Five-week-old female ICR mice were divided into five groups, each consisting of six mice. Animals were injected twice subcutaneously on the back with CNP at a single dose of 0.15, 0.3, 0.75, 1.5 mg in 0.5 mL saline solution at weekly intervals, and monitored daily for 14 days. Saline-treated animals were included as control and the toxicity was assessed by lethality, local swelling and loss of hair at the site of injection.

### 2.5. Immunization

Five-week-old female ICR mice were divided into six groups, each consisting of six mice. Animals were immunized subcutaneously with OVA (25 μg) alone or with OVA (25 μg) dissolved in saline containing QuilA (10 μg), or CS (50 μg) or CNP (12.5, 50, 200 μg) on day 1. The boosting injection was given 2 weeks later. Saline-treated animals were included as controls. Splenocytes and sera were collected 2 weeks after the secondary immunization for measurement of OVA-specific antibody, natural killer (NK) cell activity and proliferation assay.

### 2.6. Measurement of OVA-Specific IgG and Subclasses

OVA-specific IgG, IgG1, IgG2a, and IgG2b antibodies in sera were detected by an indirect ELISA. In brief, microtiter plate wells (Nunc) were coated with 100 μL OVA solution (25 μg/mL in 50 mM carbonate–bicarbonate buffer, pH 9.6) for 24 h at 4 °C. The wells were washed three times with PBS containing 0.05% (v/v) Tween 20 (PBS/Tween), and then blocked with 5% FCS/PBS at 37 °C for 2 h. After three washings, 100 μL of a series of diluted sera samples (initial dilution 1:50) or 0.5% FCS/PBS as control were added to triplicate wells. The plates were then incubated for 2 h at 37 °C, and then washed three times. Aliquots of 100 μL of rabbit anti-mouse IgG horseradish peroxidase conjugate diluted 1:10,000, goat anti-mouse IgG1 peroxidase conjugate 1:8000, IgG2a peroxidase conjugate 1:8000, and IgG2b peroxidase conjugate 1:8000 with 0.5% FCS/PBS were added to each plate. The plates were further incubated for 2 h at 37 °C. After washing, the peroxidase activity was assayed as follows: 100 μL of substrate solution (10 mg of *O*-phenylenediamine and 37.5 μL of 30% H_2_O_2_ in 25 mL of 0.1 M citrate–phosphate buffer, pH 5.0) was added to each well. The plate was incubated for 10 min at 37 °C, and enzyme reaction was terminated by adding 50 μL/well of 2 N H_2_SO_4_. The optical density was measured in an ELISA reader at 490 nm, where sets of sera samples have been subjected to within and between group comparisons, ELISA assays were performed on the same day for all of the samples.

### 2.7. Assay of Natural Killer (NK) Cell Activity

Spleen collected from the OVA-immunized mice under aseptic conditions, in Hank’s balanced salt solution (HBSS, Sigma), was minced using a pair of scissors and passed through a fine steel mesh to obtain a homogeneous cell suspension. The erythrocytes were lysed with ammonium chloride (0.8%, w/v). After centrifugation (380× *g* at 4 °C for 10 min), the pelleted cells were washed three times in PBS, and resuspended in complete medium. Cell numbers were counted with a hemocytometer by trypan blue dye exclusion technique. Cell viability exceeded 95%. The activity of NK cells was measured as previously described [36]. Briefly, K562 cells were used as target cells and seeded in 96-well U-bottom microtiter plate (Costar) at 1 × 10^5^ cells/well in RPMI 1640 complete medium. Splenocytes prepared were used as the effector cells, and were added at 5 × 10^6^ cells/well to give E/T ratio 50:1. The plates were then incubated for 20 h at 37 °C in 5% CO_2_ atmosphere. 50 μL of MTT solution (2 mg/mL) was added to each well and the plate was incubated for another 4 h and subjected to MTT assay. Three kinds of control measurements were performed: Target cells control, blank control and effector cells control. NK cell activity was calculated as the following equation: NK activity (%) = (ODT − (ODS − ODE))/ODT × 100, where ODT is the optical density value of target cells control; ODS is the optical density value of test samples; and ODE is the optical density value of effector cells control.

### 2.8. Splenocyte Proliferation Assay

Spleen collected from the OVA-immunized mice under aseptic conditions, in Hank’s balanced salt solution (HBSS, Sigma), was minced using a pair of scissors and passed through a fine steel mesh to obtain a homogeneous cell suspension, and the erythrocytes were lysed with ammonium chloride (0.8%, w/v). After centrifugation (380× *g* at 4 °C for 10 min), the pelleted cells were washed three times in PBS, and resuspended in complete medium. Cell numbers were counted with a haemocytometer by trypan blue dye exclusion technique. Cell viability exceeded 95%. Splenocyte proliferation was assayed as previously described [37]. Briefly, splenocytes from each mouse were seeded into four wells of a 96-well flat-bottom microtiter plate at 5 × 10^6^ cell/mL in 100 μL of complete medium. Con A (final concentration 5 μg/mL), LPS (final concentration 10 μg/mL), OVA (final concentration 30 μg/mL), or medium were then added, giving a final volume of 200 μL. The plates were incubated at 37 °C in a humid atmosphere with 5% CO_2_. After 44 h, 50 μL of MTT solution (2 mg/mL) was added to each well and incubated for further 4 h. The plates were centrifuged (1400× *g*, 5 min) and the untransformed MTT was removed carefully by pipetting. 150 μL of a DMSO working solution (192 μL DMSO with 8 μL 1 N HCl) was added to each well, and the absorbance was evaluated in an ELISA reader at 570 nm with a 630 nm reference after 15 min. The stimulation index (SI) was calculated based on the following formula: SI = the absorbance value for mitogen-cultures divided by the absorbance value for non-stimulated cultures.

### 2.9. Cytokine Determination in the Cultured Supernatants of Splenocytes by ELISA

Splenocytes (5 × 10^5^ cells/well) from the immunized mice prepared as described before were incubated with ConA (final concentration 5 μg/mL) in 24-well culture plates at 37 °C in 5% CO_2_. After 48 h, the plate was centrifuged at 1400× *g* for 5 min and culture supernatants were collected for the determination of INF-γ, IL-2, IL-10 levels. The presence of INF-γ, IL-2, IL-10 in the cultured supernatants of splenocytes were determined using the mouse ELISA kits (Rapidbio Lab., West Hills, CA, USA).

### 2.10. Reversed Transcript-Polymerase Chain Reaction (RT-PCR) for Cytokines Gene Expression

Splenocytes from the immunized mice prepared as described before were seeded into 24-well lat-bottom microtiter plate (Nunc) at 5 × 10^6^ cell/mL in 1 mL complete medium, then ConA (final concentration 5 μg/mL) was added giving a final volume of 2 mL (triplicate wells). The plates were incubated at 37 °C in a humidified atmosphere 5% CO_2_. After 12 h treatment, cells were collected by centrifugation (380× *g* at 4 °C for 10 min), and washed with ice-cold PBS, then subjected to RNA extraction. Cells were lysed in 0.8 mL of Trizol reagent (Invitrogen, China) and the total RNA was isolated according to the manufacture’s protocol. The concentration of total RNA was quantified by determining the optical density at 260 nm. The total RNA was used and reverse transcription was performed by mixing 2 μg of RNA with 0.5 μg oligo (dT)_18_ primer in a DEPC-treated tube. Nuclease-free water was added giving a final volume of 12.5 μL. This mixture was incubated at 70 °C for 5 min and chilled on ice for 2 min. Then, a solution containing 4 μL of M-MuLV 5×reaction buffer, 2 μL of 10 mM dNTP, 20 U of ribonuclease inhibitor, and DEPC-treated water was added, giving a final volume of 19 μL, and the tubes were incubated for 5 min at 37 °C. The tubes then received 200 U of M-MuLV reverse transcriptase and were incubated for 60 min at 42 °C. Finally, the reaction was stopped by heating at 70 °C for 10 min. The samples were stored at −20 °C until further use.

As shown in Table 1, the primers were used to amplify cDNA fragments (381-bp IL-2 fragment, 460-bp IFN-γ fragment, 324-bp IL-10 fragment and 564-bp GAPDH fragment). Amplification was carried out in total volume of 50 µL containing 4 µL (10 µM) of each cytokine-specific primers, 5 μL of 10× PCR buffer, 4 μL of MgCl_2_ (25 mM), 4 μL of dNTPs (2.5 mM), 2 µL of transcribed cDNA, and 0.25 µL of Taq DNA polymerase. PCR was performed for 33 (IL-2), 32 (IFN-γ), 30 (IL-10) or 28 (GAPDH) cycles using a MyCycler (Bio-Rad, Hercules, CA) with the following program of denaturation at 94 °C for 5 min, following by indicated cycles of 94 °C for 30 s, annealing at 58 °C (GAPDH), 60 °C (IL-2), 62 °C (IL-10), 63 °C (IFN-γ) for 30 s, and elongation at 72 °C for 30 s, and a final extension step at 72 °C for 10 min. Semi-quantitative RT-PCR was performed using GAPDH as a house keeping gene to normalize gene expression for the PCR templates. The PCR products were studied on a 1.5% agarose gel and the amplified bands were visualized using Gel DOC2000 (Bio-Rad, USA) after staining with GoldView. The size of the amplification fragments was determined by comparison with a standard DNA marker. The relative level of cytokine expression is calculated for 100 copies of the GAPDH house keeping gene following the formula: *n* = 100 × (the intensity of cytokine gene expression band/the intensity of GAPDH band).

### 2.11. Statistical Analysis

Data were expressed as mean ± standard deviations (S.D.) and examined for their statistical significance of difference with ANOVA and a Tukey post hoc test by using SPSS 16.0. *P*-values of less than 0.05 were considered statistically significant.

## 3. Results

### 3.1. Morphology, Size and Zeta Potential of CNP

**Figure 1:**
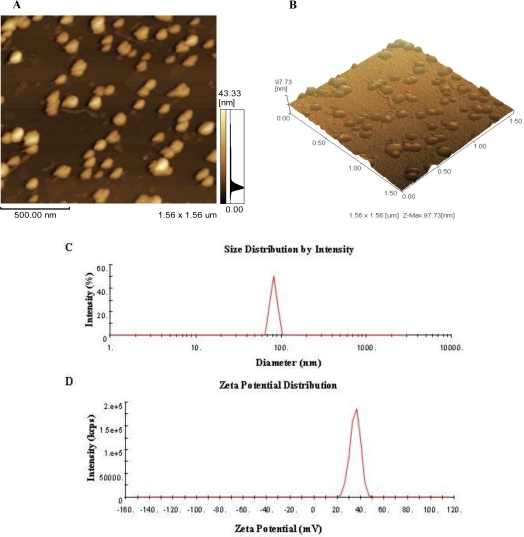
Morphology of chitosan nanoparticles (CNP). (**A**, **B**) atomic force micrographs (AFMs) of CNP; (**C**) the size distribution by intendity of CNP, the size of CNP ranges from 63.16 to 101.70 nm, and the mean of size is about 83.66 nm; (**D**) Zeta potential distribution of CNP, CNP exhibit a zeta potential range from 20.04 to 51.13 mV and have a mean charge with 35.43 mV.

As shown in Figure 1A,B, chitosan nanoparticles regularly formed and well distributed in acetic acid (HOAc)/sodium tripolyphosphate solution (pH 5.5) were used in this study. The mean size and size distribution of each batch of nanoparticle suspension was analyzed using the Zetasizer analysis (Figure 1C,D). The size distribution profile represents a typical batch of nanoparticles with a mean diameter of 83.66 nm and a narrow size distribution ranging from 63.16 to 101.70 nm (polydispersity index <1), and shows that the surfaces of chitosan nanoparticles have a positive surface charge of about 35.43 mV. These excellent characteristics are of benefit to the stabilization and penetration capability of CNP, enabling CNP to easily penetrate through capillary and epithelial tissue.

### 3.2. Toxicity of CNP

The endotoxin level in a stock CNP with a concentration of 3 mg/mL was measured to be less than 0.5 endotoxin units (EU)/mL. Therefore, the CNP samples used in this study were excluded from endotoxin contamination. When the animals were administered subcutaneously twice ranging from 0.15 to 1.5 mg at weekly intervals, no harm was observed. Local swelling or loss of hair was not observed in mice at the test doses. The results suggested that the safety dose of CNP used for animals was at least up to 60 mg/kg.

### 3.3. Effect of CNP on the OVA-Specific Serum Antibody Response

**Figure 2:**
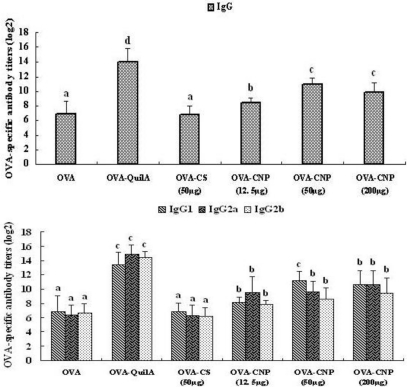
Effect of CNP on OVA-specific IgG, IgG1, IgG2a and IgG2b antibody titers in OVA-immunized mice. Mice (*n* = 6/group) were subcutaneously injected with OVA (25 μg) alone or with OVA 25 (μg) dissolved in saline containing CNP (12.5, 50 or 200 μg), CS (50 μg) or QuilA (10 μg) on days 1 and 15. Sera were collected 2 weeks after the secondary immunizations for analysis these OVA-specific antibodies using indirect ELISA. Bar with different letters are statistically different (*P* < 0.05). Abbreviations: QuilA: mixture of triterpene saponins from the bark of *Quillaja saponaria* Molina.

The OVA-specific IgG, IgG1, IgG2a and IgG2b antibody levels in the serum were measured by the indirect ELISA method two weeks after the last immunization (Figure 2). As a positive control, QuilA elicited the highest IgG and IgG isotypes levels. The serum IgG and IgG1 levels in mice immunized with OVA were significantly enhanced by CNP compared with the control groups (OVA and CS) (*P* < 0.05). Moreover, significant enhancements in OVA-specific IgG2a and IgG2b levels were observed in mice immunized with CNP compared with the OVA and CS control group (*P* < 0.05), and there were significant differences among CNP at all doses and QuilA (*P* < 0.05).

### 3.4. Effect of CNP on Natural Killer (NK) Cell Activity in OVA-Immunized Mice

**Figure 3:**
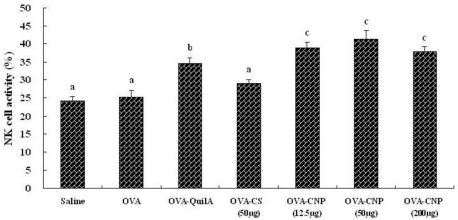
Effect of CNP on NK cell activity in mice immunized with OVA. Mice (*n* = 6/group) were subcutaneously injected with OVA (25 μg) alone or with OVA 25 μg dissolved in saline containing CNP (12.5, 50 or 200 μg), CS (50 μg) or QuilA (10 μg) on days 1 and 15. Bars with different letters are statistically different (*P* < 0.05).

The effects of CNP on natural killer (NK) cell activity in OVA-immunized mice were shown in Figure 3. CNP and Quil A significantly enhanced the killing activity of NK cell in the OVA-immunized mice (*P* < 0.05). The findings indicated that CNP could promote lytic activity of NK cells in mice immunized with OVA.

### 3.5. Effect of CNP on Splenocyte Proliferation in OVA-Immunized Mice

**Figure 4:**
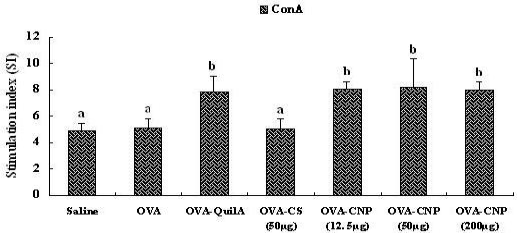

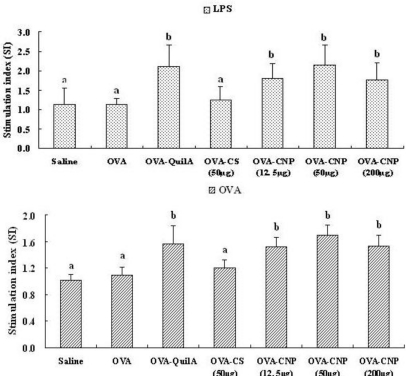
Effect of CNP on mitogen- and OVA-stimulated splenocyte proliferation in the mice immunized with OVA. Bars with different letters are statistically different (*P* < 0.05).

The effects of CNP and QuilA on splenocyte proliferative responses to ConA, LPS and OVA stimulation are shown in Figure 4. Mice immunized with OVA plus CNP or QuilA had higher splenocyte proliferative response to ConA, LPS and OVA than the mice injected with OVA alone (*P* < 0.05). Mice immunized with OVA plus CNP also had higher ConA-, LPS- and OVA-stimulated splenocyte proliferative response than the mice injected with OVA plus CS (*P* < 0.05).

### 3.6. Effect of CNP on Cytokines Level in Splenocytes from the OVA-Immunized Mice

**Figure 5:**
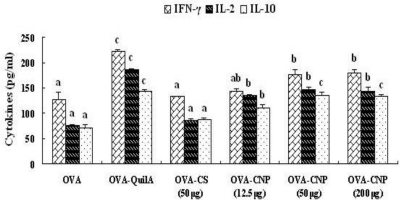
Effects of CNP on cytokine production in splenocytes from the OVA-immunized mice. Splenocytes were prepared and cultured with Con A for 48 h. The levels of IL-2, IFN-γ and IL-10 in the culture supernatants were determined by ELISA as described in the text. Values are expressed as means ± S.D. of six animals. Bars with different letters are statistically different (*P* < 0.05).

The contents of cytokines IFN-γ, IL-2 and IL-10 in the supernatants from cultured splenocytes in the mice immunized with OVA-CNP were significantly higher than those in OVA and OVA-CS control mice (*P* < 0.05) (Figure 5). QuilA enhanced significantly the production of cytokine IL-10 (Th 2 type immune response), IFN-γ and IL-2 (Th 1 type immune response) (*P* < 0.05) in the supernatants from cultured splenocytes in the mice immunized with OVA. These results suggested that CNP significantly enhanced the production of the Th1 and Th2 cytokines in OVA-immunized mice, and CNP markedly improved the adjuvant activity of chitosan in the OVA-immunized mice.

### 3.7. Effect of CNP on mRNA Expression of Cytokines in Splenocytes from the Immunized Mice

**Figure 6:**
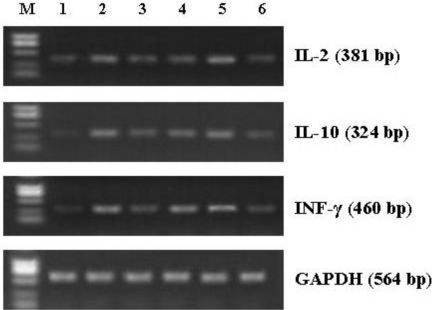
Eeffect of CNP on the mRNA expression of cytokines and GAPDH in splenocytes from the OVA-immunized mice. Lane M, DNA marker; lane 1, OVA; lane 2, QuilA; lane 3, OVA-CNP (12.5 μg); lane 4, OVA-CNP (50 μg); lane 5, OVA-CNP (200 μg); lane 6, OVA-CS (50 μg).

As shown in Figure 6 and Table 2, CNP and QuilA not only significantly increased the mRNA expression of Th1 cytokines IL-2 and IFN-γ (*P* < 0.05), but also enhanced that of Th2 cytokines IL-10 in splenocytes from the immunized mice (*P* < 0.05). Therefore, the findings suggested that CNP up-regulated the gene expression of Th1/Th2 cytokines in splenocytes from the immunized mice.

## 4. Discussion

Immunization has been the most effective way to protect individuals and the community against debilitating infectious diseases, thereby preventing the potential economic losses and morbidity associated with such diseases [25]. New generations of vaccines, particularly those based on purified recombinant proteins, synthetic peptides and plasmid DNA, despite their better tolerability, are unfortunately often much less reactogenic and immunogenic. Therefore, there is an urgent need for the development of new and improved vaccine adjuvants [38]. Although a variety of adjuvants have been used in experimental vaccines, most of these materials only elicit an antibody response and/or have undesirable side effects that have limited their potential application in vaccines [39,40].

In the previous studies, chitosan particles could activate components of the nonspecific immune system such as macrophages and NK cells, and could induce nonspecific immunity to bacteria, fungi, and tumors [28,41,42]. In addition, Chitosan particles can also activate dendritic cells (DCs) via the membrane receptors (TLR4 and mannose receptors) [43]. DCs are thought to be the most effective antigen-presenting cells (APCs) in immune response, although macrophages can also function in this role. Activated DCs lead to cytokine production, increase levels of membrane markers, such as major histocompatibility complex class II molecules, and possess the capacity to activate naive T cells. Furthermore, Chitosan micro- and nanoparticles have been reported to have immune-stimulating activity such as increasing accumulation and activation of macrophage and polymorphonuclear cell, promoting resistance to infections by microorganisms, and inducing cytokines [44]. These studies indicated that chitosan particles could stimulate macrophage, DCs, B and T lymphocytes. Therefore, the ability of chitosan nanoparticles used as immunological adjuvants to induce both humoral and cell-mediated immunity seems promising. To further research the safer adjuvant, the present study was undertaken to evaluate the toxicity of CNP and its adjuvant potential on the cellular and humoral immune responses of mice against OVA.

The cellular immune response plays an important role in the host response to intracellular pathogens by limiting replication and accelerating clearance of infected cells as well as in the generation of both humoral and cell-mediated responses to vaccination. Among the T-lymphocytes, helper T cells induce B-lymphocytes to secrete antibodies, and cytotoxic T-lymphocytes help phagocytes to destroy ingested microbes and to kill intracellular microbes. Humoral immunity, however, mediated by antibodies which are produced by B-lymphocytes, functions by neutralizing and eliminating extracellular microbes and microbial toxins. The capacity to elicit an effective T- and B-lymphocyte immunity can be shown by the stimulation of lymphocyte proliferation response. It is generally known that Con A stimulates T cells and LPS stimulates B cell proliferation [45]. We evaluated whether CNP could enhance the cellular immune responses to OVA in mice when given together with OVA. As a positive control, QuilA is known to be a powerful experimental adjuvant, and significantly elicited the mitogen- and OVA-stimulated splenocyte profilerations in OVA-immunized mice [45]. As shown in Figure 4, CNP and QuilA significantly enhanced the mitogen- and OVA-stimulated splenocyte profilerations in OVA-immunized mice as compared with OVA and CS groups, while there was no significant difference between CNP and QuilA. The proliferation assay showed that CNP could significantly promote the Con A-, LPS-, and OVA-stimulated splenocyte proliferation in the immunized mice. The results indicated that CNP could significantly increase the activation potential of T and B cells, and induce the humoral immunity and cell-mediated immune response in the OVA-immunized mice.

Evidence now exists to clearly suggest that Th1 or Th2 responses, generated upon antigenic stimulation, can be modulation *in vivo* depending on the adjuvant used for immunization [46,47]. The different Th1 and Th2 immune response profiles correspond to the activation of two distinct major subsets of T-cells characterized by their pattern of cytokine production [48]. The Th1 immune response is characterized by the production of cytokines IL-2, TNF-β and IFN-γ, and an enhanced production of IgG2a, IgG2b, IgG3 in mice. The Th2 response is characterized by the production of cytokines IL-4, IL-10 and an enhanced production of IgG1 [49]. Immunity to different infectious agents required distinct types of immune responses. The Th1 response, correlated with the induction of cell-mediated immunity [50], is required for protective immunity against intracellular infectious agents, such as certain bacteria, protozoa and presumably against cancer cells [51]. Th2 immunity, which control the humoral immune response through the triggering of B cell proliferation and differentiation [52], is effective for protection against most bacterial as well as certain viral infections [53]. In the present study, the adjuvant activity of CNP on the humoral immune responses to OVA was also evaluated. While OVA alone induced low levels of IgG, IgG1, IgG2a and IgG2b antibodies (Figure 2), the addition of CNP to OVA resulted in dramatic increase in IgG, IgG1, IgG2a and IgG2b antibody titers. Meanwhile, as a positive control group, QuilA could also increase IgG, IgG1, IgG2a and IgG2b antibody titers, resulting in a mixed Th1/Th2 immune response. Thus, in addition to enhancing the magnitude of antibody responses, CNP also modulated the quality of the immune responses, and elicited a balanced Th1/Th2 immune response to OVA in mice as indicated by the significant increases in both IgG1, IgG2a and IgG2b antibody isotypes.

In order to clearly establish that Th cell-derived cytokines were involved in the adjuvant activity of CNP, we analysed the Th1/Th2 cytokines secretion profiles in OVA-immunized mice using ELISA. CNP not only significantly enhanced the production of Th2 cytokine IL-10, but also strongly increased the production of Th1 cytokines IL-2 and IFN-γ from splenocytes in the OVA-immunized mice (Figure 5). To further elucidate the mechanism responsible for the changes in the amounts of Th1/Th2 cytokines, we utilized RT-PCR to analyse the mRNA expression of IL-2 and IFN-γ, the typical Th1 cytokines, and IL-10, the archetypal Th2 cytokine in splenocytes of the immunized mice. CNP not only enhanced the mRNA expression of IL-10, but also increased that of IL-2 and IFN-γ. Cytokines mRNA levels were positively correlated with protein expression of cytokines, *i.e.*, the levels of cytokines. In this study, CNP significantly enhanced the levels of cytokines (IL-2, IFN-γ and IL-10) in OVA-immunized mice. In mice, IL-10 preferentially switch activated B cells to the IgG1 isotype (Th2 type); IFN-γ and IL-2 enhanced IgG2a and IgG2b response (Th1 type) [54]. These results suggested that the effects of CNP on Th1 and Th2 immune response may result, at least in part, from the regulation of mRNA expression of the cytokines.

Natural killer cells (NK cells) are the type of cytotoxic lymphocyte that constitute the major component of the innate immune system. NK cells and CTL play the important role in the defense against tumors and cells infected by viruses [55–57], and moreover, represent two major populations of cytotoxic lymphocytes [58,59]. With spontaneous cell-mediated cytotoxicities, NK cells are also functionally similar to CTLs. NK cells are capable of delivering a response immediately after recognizing specific signals, including stress signals, “danger” signals or signals from molecules of foreign origin [60]. NK cells can react against and destroy a target cell without prior sensitization to it. The target cell could be a cancer cell cultured *in vitro* or from another tissue. NK cell activity assay is a routine method for analysis of a patient’s cellular immune response *in vitro*, and can also be used to test the antitumor activities of possible drugs [61]. In this investigation, as shown in Figure 3, CNP significantly enhanced the lytic activity of NK cells in OVA immunized mice, suggesting that CNP could improve cytolytic activities against autologous tumor cells and viruses.

## 5. Conclusions

Based on the findings presented herein, our data suggested that CNP has immunological adjuvant activity on the specific cellular and humoral immune responses to OVA in mice. Taking account of its natural origin and good biocompatibility, and without lethal toxicity to humans and animals, CNP may be a safe and efficacious adjuvant candidate suitable for a wide spectrum of prophylactic and therapeutic vaccines, for which a balanced and potent stimulation of both the cellular and humoral responses is required. Research on CNP with various types of antigen, including vaccines clinically used in other animal models, verify the adjuvant effect. Moreover, the mechanism of the action of CNP has still not clearly been explained. Therefore, more studies on the mechanism of the adjuvant effect of CNP are needed to elucidate in more detail.

## Figures and Tables

**Table 1:** Sequences of primer used for Reversed Transcript-Polymerase Chain Reaction (RT-PCR). GAPDH, glyceraldehyde-3-phosphate dehydrogenase.

**Gene**	**Primer sequence**	**Product size (bp)**
IL-2	5′-CTCTACAGCGGAAGCACAGC-3′ 5′-CATCTCCTCAGAAAGTCCACCA-3′	381
IFN-γ	5′-TGAACGCTACACACTGCATCTTGG-3′ 5′-CGACTCCTTTTCCGCTTCCTGAG-3′	460
IL-10	5′-CCAGTTTTACCTGGTAGAAGTGATG-3′ 5′-TGTCTAGGTCCTGGAGTCCAGCAGACTCAA-3′	324
GAPDH	5′-CCCACAGTAAATTCAACGGCAC-3′ 5′-CATTGGGGTTAGGAACACGGA-3′	564

**Table 2:** The mRNA expression level of cytokines in splenocytes from the OVA-immunized mice.

**Gene**	**OVA**	**OVA-QuilA**	**OVA-CNP**			**OVA-CS**
			**(12.5 μg)**	**(50 μg)**	**(200 μg)**	**(50 μg)**

IL-2	15.0 ± 1.4 ^a^	45.6 ± 2.1 ^c^	26.6 ± 1.4 ^b^	38.8 ± 2.1 ^bc^	50.6 ± 2.2 ^c^	16.0 ± 1.2 ^a^
IFN-γ	15.8 ± 1.6 ^a^	40.4 ± 2.3 ^c^	29.0 ± 1.4 ^b^	38.8 ± 2.3 ^bc^	44.0 ± 2.5 ^c^	16.0 ± 1.6 ^a^
IL-10	15.0 ± 1.4 ^a^	45.0 ± 2.7 ^c^	22.4 ± 1.8 ^ab^	30.4 ± 2.2 ^b^	44.8 ± 2.7 ^c^	18.2 ± 1.7 ^a^

Means within a row with different letters (a, b, c) differ significantly (*P* < 0.05).
